# Transcatheter aortic valve implantation in patient with combined aortic and subaortic membrane stenosis: a case report

**DOI:** 10.1093/ehjcr/ytae621

**Published:** 2024-11-22

**Authors:** Hesham Tayel, Abdelrahman Elhakim, Mohamed Elsoudi, Amira Nour, Mohammed Saad

**Affiliations:** Cardiology Department, Menofia University, Gamal Abd El-Nasir, Shebeen El-Kom, Menofia 6132501, Egypt; Cardiology Department, Schleswig-Holstein University Hospital-Kiel, Arnold-Heller-Street 3, 24105 Kiel, Germany; Cardiology Department, Alazhar University, Yosief Abbas Street, Cairo 11754, Egypt; Cardiology Department, Ain Shams University, 1 Elsarayat St., Abbaseya, 11517 Cairo, Egypt; Cardiology Department, Schleswig-Holstein University Hospital-Kiel, Arnold-Heller-Street 3, 24105 Kiel, Germany

**Keywords:** Aortic valve stenosis, Subaortic membrane, Transcatheter aortic valve implantation

## Abstract

**Background:**

The combination of symptomatic aortic stenosis (AS) and subaortic membrane (SAM) is rare, and the haemodynamic consequences of this combination are challenging to diagnose and manage.

**Case summary:**

We present the case of a 78-year-old male who presented with symptomatic AS and chronic heart failure. On echocardiography, the combination of AS and SAM was diagnosed. Transoesophageal echocardiography (TOE) was performed to confirm the presence and severity of AS and SAM. Based on the patient’s clinical profile and the high risk of surgical repair, a self-expandable transcatheter aortic valve implantation (TAVI) was performed. Three-month follow-up was uneventful. In addition, we discuss the assessment and management strategy challenges for SAM in patients with aortic regurgitation or AS.

**Discussion:**

As transcatheter valve implantation expands its indications for more complex anatomy and patient’s comorbidity. Self-expandable TAVI-prosthesis under TOE guidance is feasible for the treatment of symptomatic AS and SAM, especially in patients with high surgical risk.

**Learning points**

- The combination of symptomatic aortic stenosis (AS) and subaortic membrane (SAM) is rare, and the haemodynamic consequences of this combination are challenging to diagnose and manage. Differentiation of SAM from hypertrophic obstructive cardiomyopathy is of paramount importance.
- Transcatheter valve implantation expands its indications for more complex anatomy and patient’s comorbidity. Self-expandable transcatheter aortic valve implantation-prosthesis under transoesophageal echocardiography guidance is feasible for treating of symptomatic AS and SAM, particularly in patients with high surgical risk.

## Introduction

Sub-valvular aortic stenosis (SAS) is a rare genetic structural heart anomaly observed in infants. Variations in SAS that occur alone or in combination with the others are expressed as follows^1^: thin distinct membrane [subaortic membrane (SAM)], fibromuscular ridge, diffuse fibromuscular tunnel-like narrowing of the left ventricle outflow tract (LVOT), and accessory or anomalous mitral valve tissue.

Clinical presentation is rare at birth and infancy. However, in some paediatric patients, SAM may progress at a faster and unpredictable rate and develop mostly aortic regurgitation (AR).^2^

On the other hand, in adult patients, SAM has a gradual course and over time, and patients develop symptoms such as angina and heart failure.^1^

The diagnosis of SAM can be challenging because it can mimic hypertrophic obstructive cardiomyopathy (HOCM), bicuspid aortic valve, or rheumatic heart disease.

Subaortic membrane management in paediatric patients is challenging because of the potential recurrence rate of 17% and the risk of complications if untreated, SAM can worsen AR or aortic stenosis (AS).^3^

The AHA/ACC guidelines on the management of SAM recommend surgical intervention for paediatric patients with a Doppler mean gradient > 50 mmHg or with a Doppler mean gradient 30–50 mmHg may be indicated for surgery if symptomatic or asymptomatic with electrocardiogram changes at rest.^4^

Surgical management in paediatric patients can be based solely on membrane resection whereas others consider for additional septal myectomy.

A recent study of 107 patients concluded that the routine use of myectomy with membrane excision did not result in a lower rate of reoperation or higher rates of complications compared with historical controls.^5^

## Summary figure

**Figure:**
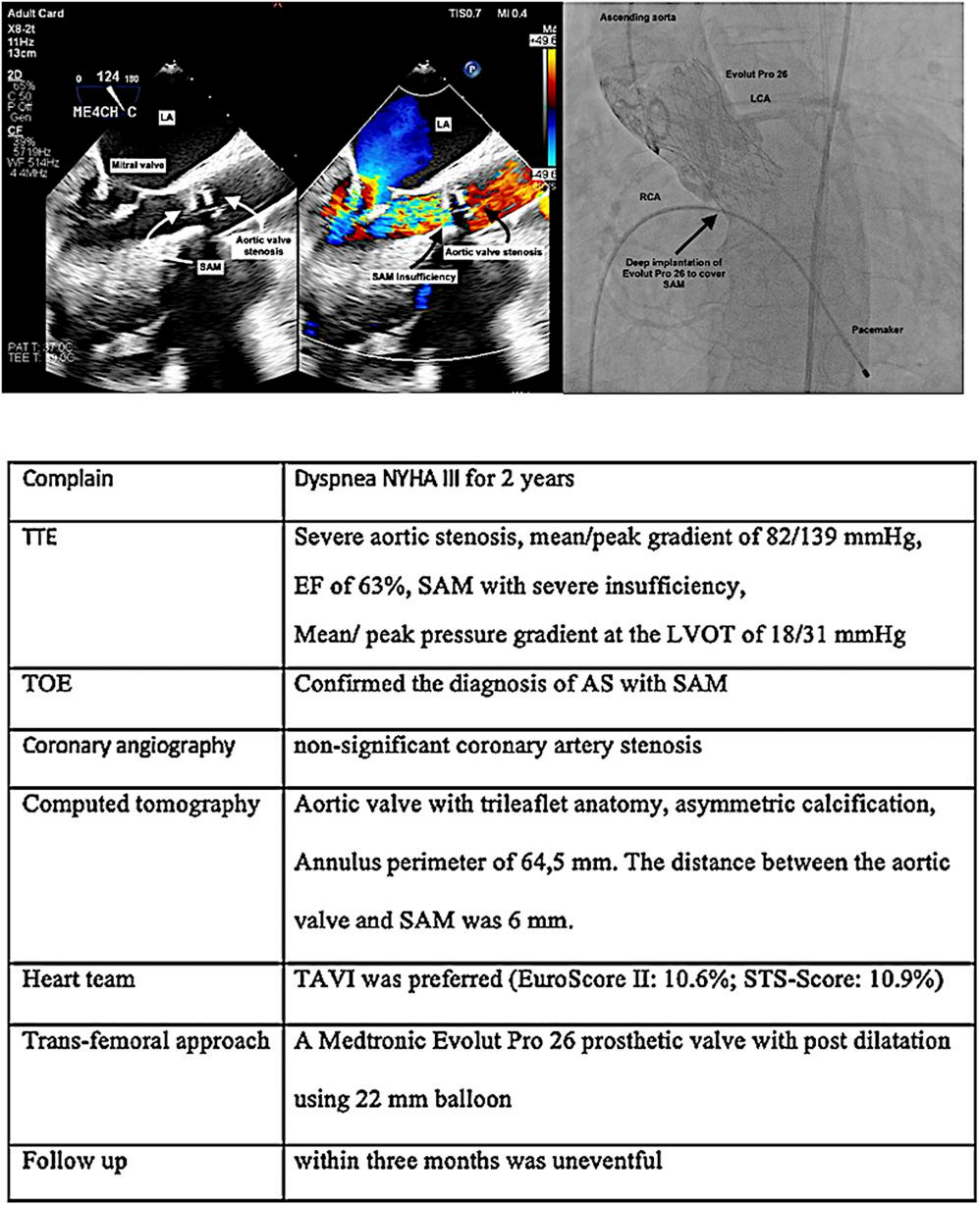


## Case presentation

A 78-year-old white male with a history of chronic renal failure initially presented with shortness of breath at rest and exertional angina for 2 years. On physical examination, a holo systolic murmur was noted, which was best heard at the right second intercostal space.

**Figure 1:**
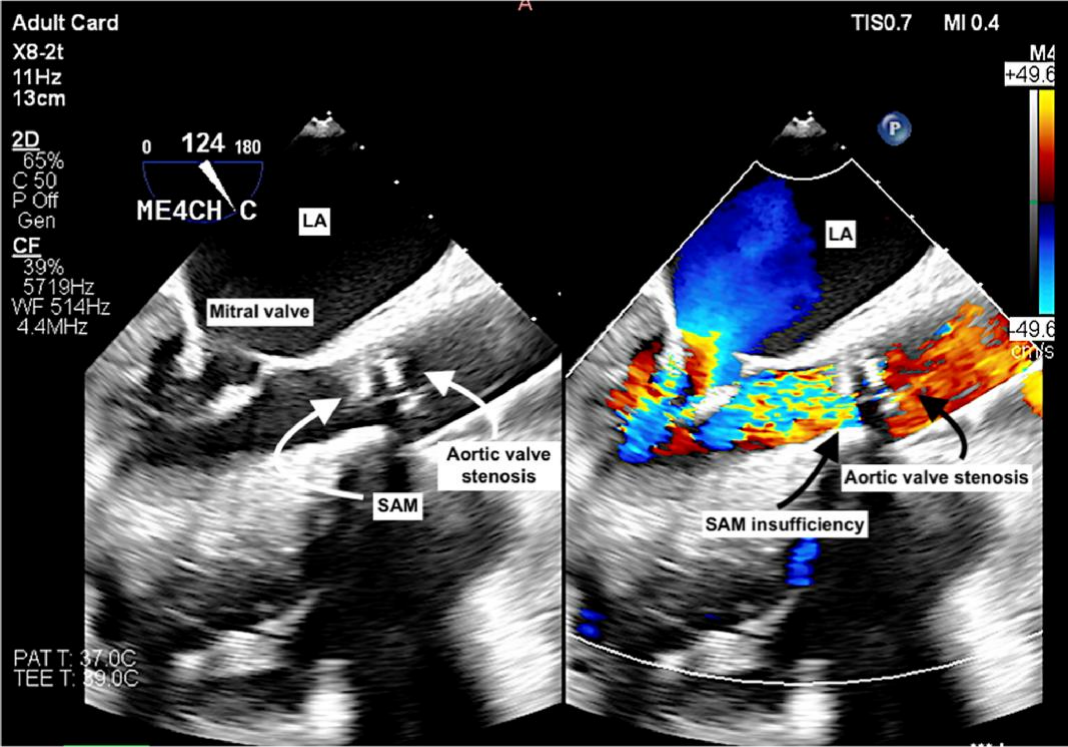
Transoesophageal echocardiography with and without colour Doppler demonstrating the presence of a subaortic membrane causing regurgitant blood flow through the aortic valve.

**Figure 2:**
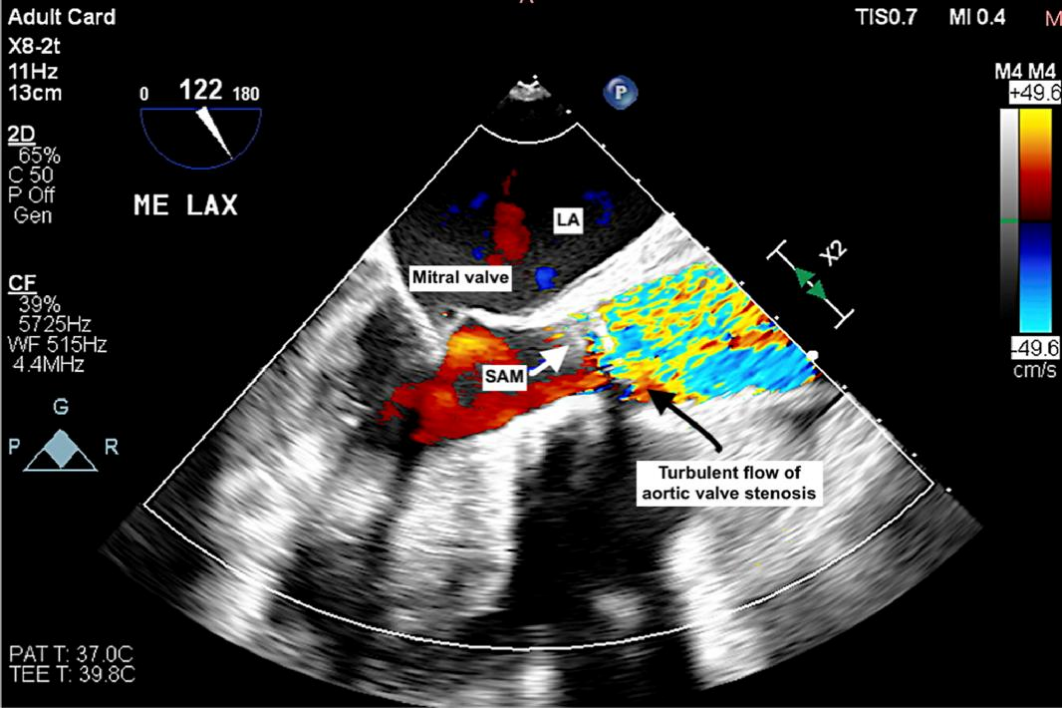
Transoesophageal echocardiography with colour Doppler demonstrating the presence of a turbulent blood flow through the aortic valve.

**Figure 3:**
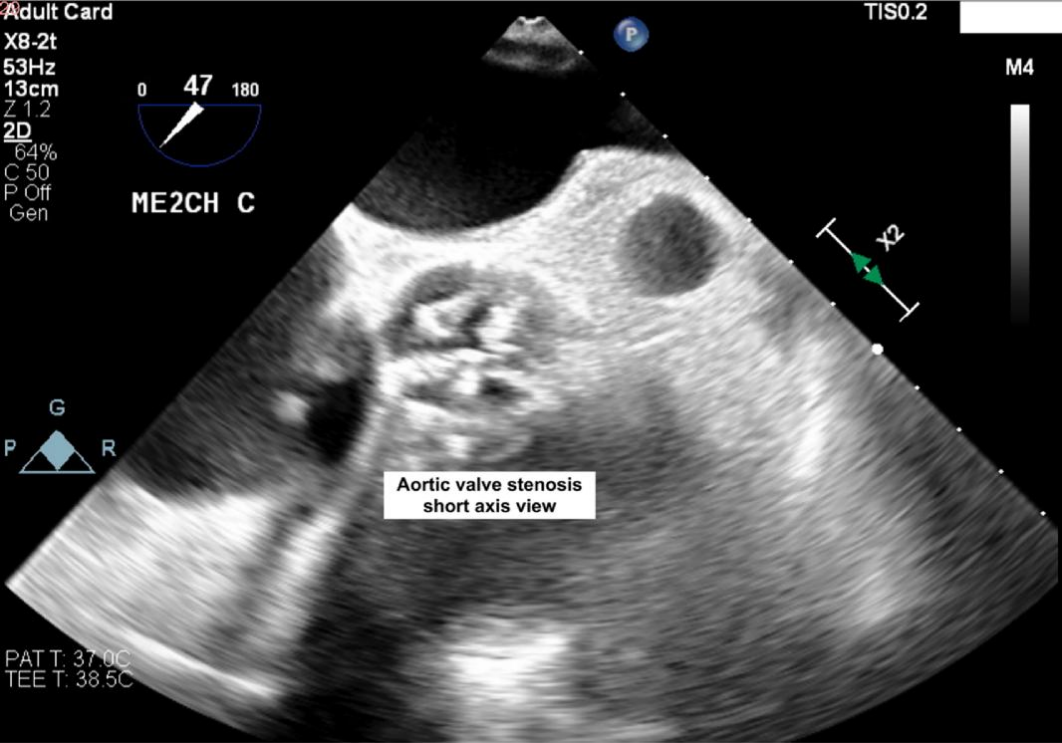
Transoesophageal echocardiography in short axis view without colour Doppler demonstrating the presence of a highly calcific and stenotic aortic valve.

A transthoracic echocardiogram revealed calcified severe AS with a mean/peak gradient of 82/139 mmHg, SAM with severe insufficiency, and an EF of 63%. Mean/peak pressure gradient at the LVOT of 18/31 mmHg. Transoesophageal echocardiography (TOE) confirmed the diagnosis (*Figures 1–3*) (see Supplementary material online, *Table S1*).

The patient underwent diagnostic catheterization, which revealed non-significant coronary artery stenosis. Due to known chronic kidney disease, we could not perform computed tomography. The TOE revealed an aortic valve with trileaflet anatomy, asymmetric calcification of the non-coronary cusp with an annulus perimeter of 64.5 mm. The distance between the aortic valve and SAM was 6 mm.

After evaluation by the heart team, transcatheter aortic valve implantation (TAVI) was preferred (EuroSCORE II: 10.6%; STS score mortality: 10.9%), and a TAVI procedure through the trans-femoral approach was selected with consensus of the heart team after extensive meticulous planning of the procedure and after patient’s consent.

We performed the procedure following the same technique as that used for the right trans-femoral route. Then, the guidewire was switched to a 0.035 inch Amplatz super stiff wire (Boston Scientific, Marlborough, USA) positioned at the apex of the left ventricle. A Medtronic Evolut Pro 26 prosthetic valve (Medtronic, Minneapolis, MN, USA) (annulus diameter 20–23 mm, annulus perimeter 62.8–72.3 mm, and skirt length 13 mm) was inserted through the aortic valve.

**Figure 4:**
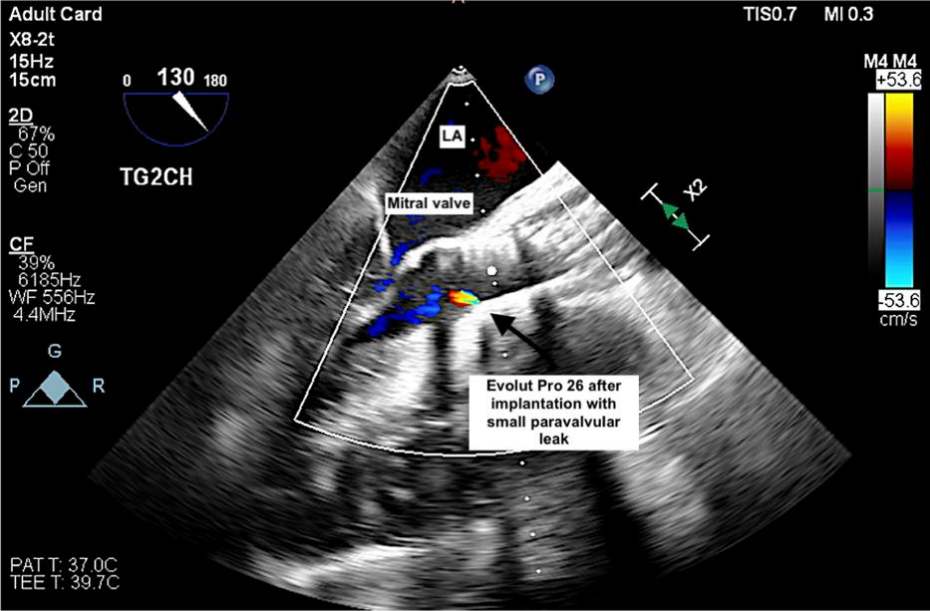
Transoesophageal echocardiography with colour Doppler demonstrating the deep implantation of Evolut Pro 26 in the aortic and SAM position without turbulent blood flow through the SAM and aortic valve, freely mobile anterior mitral leaflet and the presence of a small paravalvular leak.

**Figure 5:**
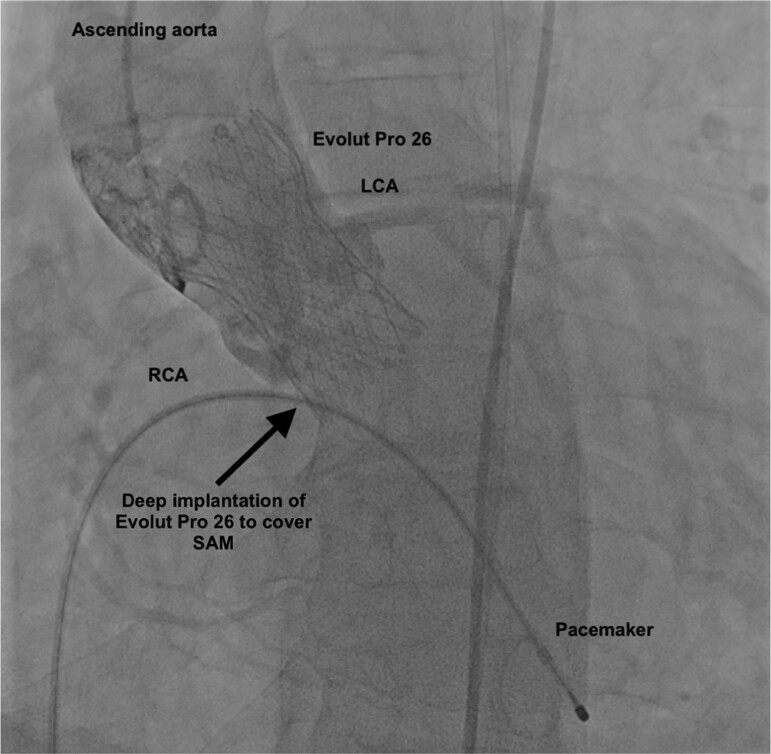
Final aortography after post-dilatation demonstrating the deep implantation of Evolut Pro 26 in the aortic and SAM position without compromising the coronary blood flow, aortic insufficiency, or paravalvular leak.

After confirming the position of the prosthetic valve with sufficient depth to cover the SAM under TOE guidance (see Supplementary material online, *Videos S1* and *S2*), the valve was partially deployed. The depth and SAM coverage were checked using TAVI-prosthesis then a further full deployment at a pacing rate of 120/min was performed. Post-dilatation was performed using 22 mm balloon (*Figures 4* and *5*). At the end of the procedure, acceptable haemodynamics (Pmean/max 7/12 mmHg, no regurgitation and small leak) in the echocardiography were delivered, and haemostasis of the femoral artery was achieved via manual compression without complications. The patient was discharged after 3 days. The 3-month follow-up was uneventful. To the best of our knowledge, this is the third reported case of successful treatment of severe valvular aortic stenosis combined with SAS and insufficiency due to SAM with TAVI.

## Discussion

We report a case of successful trans-femoral TAVI performed in a patient with combined AS and SAM insufficiency and stenosis.

Although AS is a common valvular disease in older populations, SAM is rare. The combination of these two conditions is rare, and the haemodynamic consequences of combined lesions are challenging to diagnose and manage.^6^

Transthoracic echocardiogram and TOE play crucial roles in identifying the membrane’s anatomy, the severity of LVOT obstruction, and structural damage to the aortic valve. First, it is important to diagnose AS, SAM, or a combination of these conditions. A high subaortic velocity (generally >1.5 m/s) in pulsed-wave Doppler assessment is often initially recognized.

Second, the differentiation of SAM from HOCM is important. Subaortic membrane is fixed LVOT obstruction (parabolic subaortic Doppler pattern) compared with dynamic LVOT obstruction (mid- to late-peaking, dagger-like Doppler pattern) observed in HOCM.^7^

In patients with AS and SAM, continuous-wave Doppler may reveal a double-envelope pattern, with the inner envelope representing the gradient at the SAM and the outer envelope representing the peak velocity across the aortic valve.^7^

Invasive catheterization is indicated when non-invasive assessment is uncertain.^7^

A self-expandable TAVI-prosthesis in a 78-year-old male with great surgical risk TAVI was preferred based on Recheck, and recapture and reposition features to assess AS and SAM coverage to avoid anterior mitral leaflet overhand during implantation.

Evolut 26 Prosthesis skirt with a length of 13 mm allows deep prosthesis implantation.^8^ The external wrap (Pro) provides advanced sealing and minimizes the risk of paravalvular leakage, and supra-annular prostheses will function as annular prosthetic valve function in the case of deep implantation.

A major limitation of TAVI in SAM is the distance of the SAM to the aortic valve, the deep implantation increases the risk of peacemaker indication, and prohibits SAM recurrence after TAVI is unknown, sinus of Valsalva mean diameter and height should be more than 27 and 15 mm, respectively, and the coronary artery height should be more than 10 mm.

Transcatheter aortic valve implantation with an annular valve feature was excluded, as it does not allow deep implantation, risks paravalvular leakage due to the short skirt, risk of pop-in, and inability to reassess during implantation, as it is a one-shot implantation.

Although surgical resection remains the definitive treatment, we emphasize that the trans-femoral TAVI approach is feasible, with technical considerations, as an alternative less invasive procedure, in contrast to surgical repair or replacement, in patients with combined AS and SAM.

To the best of our knowledge, this is the third report on AS and SAM treated with TAVI. Witzke^9^ reported a case of SAM combined with AR treated with a 29 mm Evolut Pro+.

Finkelstein *et al*.^10^ reported a case of severe AS and SAM treated with a 23 mm CoreValve.

However, more of cases are required to evaluate the safety and feasibility of this approach in this group of patients.

## Conclusion

The combination of symptomatic AS and SAM is rare, and its haemodynamic are challenging to diagnose and manage.

As TAVI expands its indications for more complex anatomy and patient’s comorbidity, self-expandable TAVI-prosthesis under TOE guidance is feasible for the treatment of these combined pathologies, especially in patients with high surgical risk.

## Supplementary Material

ytae621_Supplementary_Data

## Data Availability

All data related to the case are available on request. The paper is not under consideration elsewhere. None of the paper’s contents have been previously published. All authors have read and approved the manuscript. Author takes full responsibility for the content of the publication.

## Lead author biography

Fifteen years in cardiology felled, interventional cardiology consultant in Menoufia University and El-Araby Hospital in Egypt, finished my intervention cardiology fellowship in Bezmialem Vakıf Üniversitesi, İstanbul, Türkiye.

## Notes

**Consent:** Consent to participate is obtained. Written informed consent was obtained from the patient in line with COPE for publication of this case report and any accompanying images. A copy of the written consent is available for review by the Editor-in-Chief of this journal.

**Funding:** The authors received no financial support for the publication of this article.
