# Supplementary material for: Transcatheter aortic valve implantation in patient with combined aortic and subaortic membrane stenosis: a case report
Source: Eur Heart J Case Rep. 2024 Nov 22;8(12):ytae621. doi: 10.1093/ehjcr/ytae621 (PMC11649379; doi:10.1093/ehjcr/ytae621)
Supplement: ytae621_Supplementary_Data [file ytae621_supplementary_data.zip › Supplementary table.docx]

**Supp. table 1**: Summery of trans esophageal echocardiographic and duplex sonographic parameters.

| **TOE Parameter** | |
| --- | --- |
| Aortic valve area planimetry | 0,58 cm2 |
| Aortic valve peak velocity | 5,8 m/s |
| Aortic valve mean/peak gradient, | 82/139 mmHg |
| Left ventricular outflow tract (LVOT) mean/peak velocity | 18/31 mmHg |
| Aortic annulus area | 300 cm2 |
| Aortic annulus dimension | 22 and 18 mm |
| Aortic annulus perimeter | 64,5 mm |
| LVOT dimension | 21,14 mm |
| LVOT area | 2,7 cm2 |
| Subaortic membrane insufficiency   - vena contracta, - regurgitation volume - Regurgitation fraction (%) | 7 mm  >65 mls/beat  55% |
| SAM length | 12 mm |
| Obstruction of LVOT area (by area subtraction) | 51% |
| Aortic sinus diameter | 34 and 29 mm |
| Sinus-tubular junction | 30 mm |
| Ascending Aorta | 40 mm |
| Right and left coronary height | 12 and 8 mm respectively |
| Tricuspid insufficiency grad III   - vena contracta - systolic pulmonary artery pressure | 8 mm  61 mmHg |
| **Arterial Duplex** | |
| Common iliac artery right and left | 6,5 and 6,6 mm respectively |
| External iliac artery right and left | 6,3 and 6,4 mm respectively |
| Common femoral artery right and left | 6,2 and 6,2 mm respectively |
